# ECM-LSE: Prediction of Extracellular Matrix Proteins Using Deep Latent Space Encoding of k-Spaced Amino Acid Pairs

**DOI:** 10.3389/fbioe.2021.752658

**Published:** 2021-10-14

**Authors:** Ubaid M. Al-Saggaf, Muhammad Usman, Imran Naseem, Muhammad Moinuddin, Ahmad A. Jiman, Mohammed U. Alsaggaf, Hitham K. Alshoubaki, Shujaat Khan

**Affiliations:** ^1^ Center of Excellence in Intelligent Engineering Systems, King Abdulaziz University, Jeddah, Saudi Arabia; ^2^ Electrical and Computer Engineering Department, King Abdulaziz University, Jeddah, Saudi Arabia; ^3^ Department of Computer Engineering, Chosun University, Gwangju, South Korea; ^4^ Research and Development, Love For Data, Karachi, Pakistan; ^5^ School of Electrical, Electronic and Computer Engineering, The University of Western Australia, Perth, WA, Australia; ^6^ College of Engineering, Karachi Institute of Economics and Technology, Korangi Creek, Karachi, Pakistan; ^7^ Department of Radiology, Faculty of Medicine, King Abdulaziz University, Jeddah, Saudi Arabia; ^8^ Department of Bio and Brain Engineering, Daejeon, South Korea

**Keywords:** extracellular matrix (ECM), auto-encoder, composition of k-spaced amino acid pair (CKSAAP), latent space learning, neural network, classification, amino acid composition (AAC)

## Abstract

Extracelluar matrix (ECM) proteins create complex networks of macromolecules which fill-in the extracellular spaces of living tissues. They provide structural support and play an important role in maintaining cellular functions. Identification of ECM proteins can play a vital role in studying various types of diseases. Conventional wet lab–based methods are reliable; however, they are expensive and time consuming and are, therefore, not scalable. In this research, we propose a sequence-based novel machine learning approach for the prediction of ECM proteins. In the proposed method, composition of k-spaced amino acid pair (CKSAAP) features are encoded into a classifiable latent space (LS) with the help of deep latent space encoding (LSE). A comprehensive ablation analysis is conducted for performance evaluation of the proposed method. Results are compared with other state-of-the-art methods on the benchmark dataset, and the proposed ECM-LSE approach has shown to comprehensively outperform the contemporary methods.

## 1 Introduction

Extracelluar matrix (ECM) is a network of fibrous proteins filled in the extracellular spaces of living tissues to provide structural support for the cells (Karagöz et al., 2021). It is significant for cell functionality and plays an important role in the physiological dynamics. ECMs are also responsible for the promotion of vital cellular processes, including differentiation, adhesion, proliferation, apoptosis, and migration (Klavert and van der Eerden, 2021; Hiraki et al., 2021; Mathews et al., 2012; Endo et al., 2012; Kim et al., 2011). The chemical composition of ECM mainly consists of minerals, proteoglycans, proteins, and water. The proteins in ECM act more like a fibrous material which gives strength to the cells. Several studies have demonstrated that the mutation in the ECM genes can cause severe adverse effects in the cell structure resulting in a number of diseases, including arthritis and cancer (Kizawa et al., 2005; Hu et al., 2007).

Functional research on ECM protein has resulted in the development of useful biomaterials which are used in many fields of medicine, such as tissue engineering and cell therapy (Ma et al., 2019; Gonzalez-Pujana et al., 2019). Proteins, in general, are active elements and play a variety of roles depending on their residing location in a cell. Likewise, the functionality of the ECM varies with the change in the proteins. The problem of protein localization is therefore considered to be an important step toward the understanding of protein functionality (Horton et al., 2007). Identification of subcellular location is however considered to be a nontrivial task and requires extensive experimentation which is prohibitively expensive. Therefore, a variety of computational methods have been developed to facilitate the process (Ras-Carmona et al., 2021; Wang et al., 2021; Chou, 2011). In particular, for different species of plants, animals, and microorganisms, a number of useful techniques have been explored (Zhao et al., 2021; Hou et al., 2021; Chou et al., 2012; Otzen et al., 2021; Wu et al., 2011; Asim et al., 2021; Xiao et al., 2011; Shen et al., 2021; Wu et al., 2012; Lewis et al., 2014). Bioinformatics methods, with the aid of machine learning algorithms, have demonstrated adequate performance for a variety of applications. A detailed review of computational methods to classify secreted proteins has been provided by Klee and Sosa (2007). Typically, three aspects are focused on the development of a computational method: 1) feature extraction—in which the peptide sequence is translated/encoded into a numerical format to make them readable by the model, 2) feature selection—which is concerned with the removal of the redundant information from the feature space and results in the model’s robustness, and 3) model construction and evaluation—which includes development of a prediction model, followed by training and testing steps to evaluate performance.

The first benchmark *in-silico* approach to predict the extracellular proteins was presented by Jung et al. (2010)and was named as ECM protein prediction (ECMPP). The research used the feature augmentation method and crafted a feature set of 91 attributes. One of the limitations of the study was the use of a small dataset for performance evaluation; also, only the receiver-operating characteristics (ROC) were used for the performance evaluation. Since then, many researchers have paid attention toward the development of machine learning methods for ECM prediction. As extracellular matrix proteins are linked to the outer surface of the cell, they have close association with its secretory mechanism and are naturally associated with the secretary proteins. Therefore, it is reasonable to consider possible ECM candidates as a subset of secretary proteins (Kandaswamy et al., 2010; Bendtsen et al., 2004; Horton et al., 2006). Based on this knowledge, Kandaswamy et al. (2013) improved the ECM prediction method and presented EcmPred. EcmPred (Kandaswamy et al., 2013) used a random forest (RF)–based classifier which was trained on the combination of sequence-derived properties of the proteins including individual and group frequencies of amino acids with the physicochemical properties. Another method named prediction of ECM (PECM) (Zhang et al., 2014) utilized a handcrafted feature set designed by the combination of the most discriminative attributes of the protein sequences including evolutionary and structural information as well as the physicochemical properties of the peptide sequences. An incremental feature selection (IFS) method was employed for the selection of optimal features which were used to train a support vector machine (SVM)–based classifier. Several other methods have also been proposed to serve the task of ECM prediction. None of them, however, focuses on the encoding of sequence-driven feature into a classifiable latent-space (LS). The primary objective of latent space–based learning is to design a reduced feature space for clustering of proteins. The LS is, therefore, a representation of the input signal in a reduced space. The latent-space encoding (LSE) is based on an assumption of a low-rank input (i.e. highly redundant) which can be compressed to a low dimensional signal using LSE. The process is considered to be reversible as the original signal could be reconstructed from the LS. The details of LS and LSE have been provided in the Subsection 2.4.

Development of a feature space and selection of the best features are fundamental steps in designing machine learning models (Lyu et al., 2021). In particular, for the protein sequence classification task, a variety of feature extraction techniques have been proposed including amino acid composition (AAC), dipeptide composition (DPC), N-segmented sequence features, physicochemical composition, and secondary structure features (Naseem et al., 2017; Khan et al., 2018; Kandaswamy et al., 2011). The sole purpose of each feature extraction technique is to encode maximum useful information from a variable length protein sequence into a fixed-sized vector. In the recent past, inspired by the success of deep long short-term memory (LSTM) models, some approaches similar to word2vec (Mikolov et al., 2013) have been proposed to successfully learn latent space encoding directly from variable length sequences (Ding et al., 2019). The direct sequence to latent space encoding method produces good generalization models (Zemouri, 2020); however, they usually rely on the availability of a large training dataset. Furthermore, the direct extraction of latent space features from a limited number of sequences such as, bioluminescence (Zhang et al., 2021), antioxidant (Olsen et al., 2020), ECM (Kabir et al., 2018), antifreeze proteins (AFPs) (Kandaswamy et al., 2011), or other classes of proteins is a challenging problem. In this study, we propose a hybrid approach where all proteins are first encoded into a large feature set obtained through composition of *k*-spaced amino acid pair encoding. A latent space representation of composition of *k*-spaced amino acid pairs (CKSAAP) is learned which can help to design a robust classifier. This eliminates the need for separately developing the classifier and the feature extraction modules, and a stand-alone model effectively learns the distinguishing characteristics of classes on a lower dimensional feature space.

The rest of the article is organized as follows: the classification framework of the proposed method is presented in Section 2, followed by the extensive experimentation and discussion in Section 3, and the study is concluded in Section 4.

## 2 Methods

### 2.1 Evaluation Metrics

For proper evaluation of the proposed model, a number of standard performance metrics have been used. The most intuitive performance measure is accuracy; however, for a highly imbalanced dataset (which is the case here), accuracy is not reflective of true performance. Therefore, various evaluation parameters, such as sensitivity, specificity, and Matthew’s correlation coefficient (MCC) are reported. Youden’s index and balanced accuracy are also considered to be important evaluation metrics for imbalanced data and are, therefore, extensively explored in this research.

### 2.2 Dataset

To design the proposed method, we used the benchmark dataset provided in Kandaswamy et al. (2013). The dataset consists of 445 ECM proteins and 3,327 non-ECM proteins. The 445 ECM proteins were curated from Swiss-Prot release 67 by first filtering 1103 ECM proteins from the pool of 17,233 metazoan-secreted protein sequences. Similarly, the negative dataset of 16,130 proteins were curated from secretory proteins that are annotated as non-ECM. Later, 445 ECM and 4,187 non-ECM nonhomologous sequences were further filtered out with the help of a clustering method (Li et al., 2001) by removing the sequences which showed 70*%* or higher similarity.

### 2.3 Feature Extraction

#### 2.3.1 Composition of K-Spaced Amino Acid Pairs

One of the fundamental steps in designing a machine learning approach is the transformation of protein sequences to a numerical format. Several methods of this transformation exist and the resultant encoded vectors of the sequences are treated as the features. The common approach practiced by several researchers is to acquire various features of the same sequence by employing different encoding schemes, and their combination is utilized for training the machine learning algorithm. This laborious approach has resulted in the performance enhancement of some classifiers (Yu and Lu, 2011; Xiaowei et al., 2012; Yang et al., 2015; Xiao et al., 2016); however, some recent studies show that utilizing a single expedient-encoding scheme such as CKSAAP, which captures both short- and long-range interaction information between residues along the sequence, can result in an equally improved classification performance (Ju and Wang, 2018; Chen et al., 2019; Usman and Lee, 2019).

**FIGURE 1:**
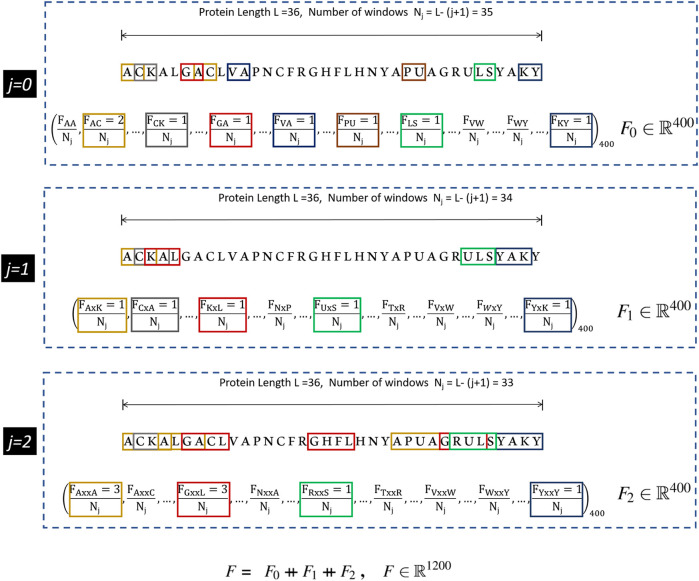
CKSAAP feature extraction mechanism for *k* = 2. Extracted from Usman et al. (2020).

The CKSAAP scheme works on the simple principle of counting the occurrence frequencies of *k*-spaced amino acid pairs in the protein sequence. Each *k*-spaced amino acid pair represents the residue pair separated by any arbitrary number *k* (*j* = 0, 1, 2 … *k*) of amino acid residues. For *k* = 0, the encoding is similar to the DPC, in which protein sequence of 20 types of amino acids yields a feature vector of (20 × 20) = 400 types of amino acid pairs (*i*.*e*., *AA*, *AC*, *AD*, … *YY*)_400_. In earlier studies it has been suggested that the DPC and higher-order peptide features can be used to design a robust protein sequence classifier (Kandaswamy et al., 2011; Khan et al., 2018; Pratiwi et al., 2017). From Figure 1, it can be seen that for higher values of *k*, substantial neighborhood information is gathered for large peptide pairs. For instance *k* = 2, three feature segments, each having a length of 400, are obtained. These are then concatenated to get the final feature vector of length (*k* + 1) × 400. The graphical representation of the CKSAAP feature vector obtained with *k* = 2 has been depicted in Figure 1.

This efficient method of encoding has, therefore, been favored by a number of researchers in various applications of computational biology including the prediction of anticancer peptides (Li et al., 2020), DNA, and several other binding sites (Ju and Wang, 2020; Lyu et al., 2020). Many adaptations of CKSAAP encoding scheme have utilized only the features generated by a single *k* value. In this research, we aim to find the optimal value of *k* by analyzing different combinations of the features generated by CKSAAP, and details are presented in Subsection 3.1.

### 2.4 Latent Space Learning for ECM Classification

Feature representation ability of the CKSAAP improves with large values of the parameter *k*, which is expected to result in a more robust model (Park et al., 2020b; Usman and Lee, 2019; Wu et al., 2019; Chen et al., 2017). However, the model utilizing a large number of features is susceptible to noise, resulting in a degraded performance. Furthermore, training the model on a large number of features not only results in an increased training time and complexity but is also prone to overfitting. To which end, feature selection/engineering, which involves the selection of most significant features, has to be employed. Feature selection techniques are broadly categorized into two types: 1) supervised methods, which remove the irrelevant features based on a target variable, and 2) unsupervised methods, which use correlation techniques to remove redundant information. A number of methods for feature selection have been proposed in the literature, including minimum redundancy maximum relevance (mRMR) (Peng et al., 2005), student’s t test (Student, 1908), info-gain (Mitchell et al., 1997), and generalized variant of strictly standardized mean difference (GSSMD) (Park et al., 2020a). Another useful method is to map the original data into a lower-order dimensional space through some transformation function. The eigen-space transformation or the principal component analysis method (PCA) (Jolliffe, 1986) is considered to be the benchmark method in this context. Other approaches such as an independent component analysis (ICA) (Comon, 1994), a kernel principal component analysis (KPCA) (Schölkopf et al., 1998), uniform manifold approximation and projection (UMAP) (McInnes et al., 2018), and t-distributed stochastic neighbor embedding (t-SNE) (Van der Maaten and Hinton, 2008) are also being successfully used to deal with the curse of dimensionality.

**FIGURE 2:**
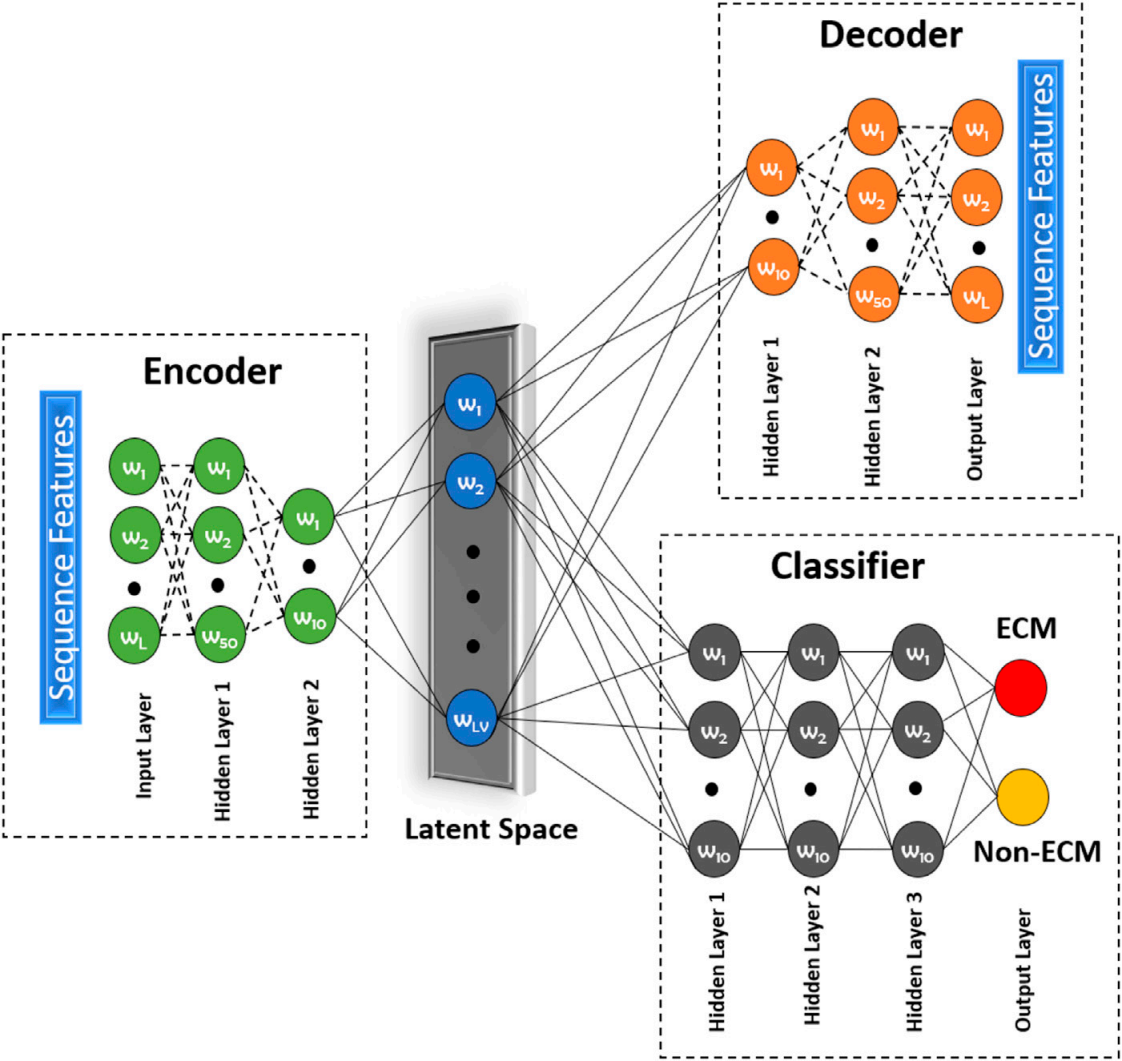
Proposed DeepLSE architecture for ECM classification. The model comprises of an encoder, a decoder, and a classifier module. The encoder consists of an input layer and two hidden layers that embed input features to latent variables (*LVs*). The decoder architecture is mirror symmetry of the encoder which uses *LVs* as its input and generates the decoded output. The classification module uses latent space features as its input and four layers of fully connected neurons. Each hidden layer has 10 neurons, except for the last layer which produces a one-hot–encoded output of ECM/non-ECM class.

Most of the techniques mentioned above are unsupervised in nature. To address this issue, we propose to use a novel approach called a deep latent space encoding (DeepLSE) classifier for the latent space encoding based on an auto-encoder. Latent space refers to the representation of compressed data in which similar points would be in a close group, as shown in Figure 5. Similar samples tend to have common significance, which can be packaged into the latent space representation of the raw data. Thus, as the dimensions are reduced, the redundant information from the input samples is removed, leaving only the most important features of the data. In other words, the method can learn a compact representation of feature space and remove the noisy or potentially confusing information which is good for both the classification and reconstruction tasks. This ensures that the encoded features truly represent the sample information. The DeepLSE method has been found to be an impressive method for the feature space reduction and has outperformed other approaches in relatively similar tasks such as AFP-LSE (Usman et al., 2020) and E3-targetPred (Park et al., 2020b). The architecture of the proposed method is depicted in Figure 2 named as ECM-LSE.

#### 2.4.1 Network Specifications

The architecture of the proposed ECM-LSE network is composed of two modules: 1) an auto-encoder module and 2) a classification module.

##### 2.4.1.1 Auto-Encoder Module

The auto-encoder is a type of neural network that can act as an identity function. It is used to find the representation of the input signal in a reduced dimensional space, known as the latent space. The principle of latent space–based representation is an assumption that the input signal has a low-rank. The auto-encoder network has a decoder that tries to regenerate the input from the latent space variables. During the training of an auto-encoder, the model is forced to become an identity function. Due to which only the relevant features of the data are learned in a compressed representation. This compressed representation has sufficient information for accurate reconstruction of the original input signal. The number of hidden layers and the number of neurons in each layer of the encoder and decoder are varied to obtain reasonable performance. In this research, the encoder and decoder are composed of three layers each, including two hidden layers. The number of neurons in the input layer of the encoder is equal to the length of the attribute vector. The number of neurons in the first and second hidden layers is set to be 50 and 10, respectively. The decoder is a mirror symmetry of the encoder. The number of neurons in the output layer of the decoder is equal to the length of the attribute vector. The number of neurons in the latent space is systematically altered to obtain the best performance for which we designed an ablation study discussed in Section 3.1. All hidden layers of the auto-encoder module are equipped with batch normalization, 30*%* dropout, and a rectified linear unit (ReLU) activation function. The latent space layer uses sigmoid activation function without any batch normalization and dropout.

##### 2.4.1.2 Classification Module

The output of the encoder module (latent variables) is used as an input to the classification module. The classifier module shown in Figure 2 consists of four layers (three hidden and one output layer). All hidden layers consist of 10 neurons and a ReLU activation function. The last layer consists of two neurons representing the positive (ECM) and the negative (non-ECM) classes. For decision making, softmax activation function was used at the output layer.

## 3 Results

To develop a neural network model, the benchmark dataset was divided into the train, validation, and test datasets. For training, we formed a dataset consisting of 540 samples with equal number of ECMs and non-ECM protein samples. These were randomly selected from the pool of 445 ECMs and 3,327 non-ECMs, since the available dataset is very small, and it is highly likely that the model would suffer from the overfitting problem. To avoid such situation, we employed regularization techniques such as early stopping, dropout, batch normalization, and DeepLSE-based feature encoding. Furthermore, the validation dataset was also used with the aim of designing a generalized classifier module. The validation dataset consists of 30 ECMs and 810 non-ECMs randomly selected from the remaining 175 ECMs and 3,182 non-ECMs, respectively. The remaining 145 and 2,247 samples of ECMs and non-ECMs were used in the test dataset. Several model configurations on the basis of the latent space size (*LVs*) and the CKSAAP gap value *k* were evaluated. For each choice of model configuration, the process of model training was repeated 20 times and mean and standard deviations of performance statistics were reported. In each trial, the weights and bias of the model were randomly initialized. Also, each trial utilized randomly configured subsets from the training, validation, and test dataset. The validation process assisted toward the filtration of the overfitted models, that is, only the models with 75*%* or higher validation balanced accuracy was selected.

### 3.1 Ablation Study

**FIGURE 3:**
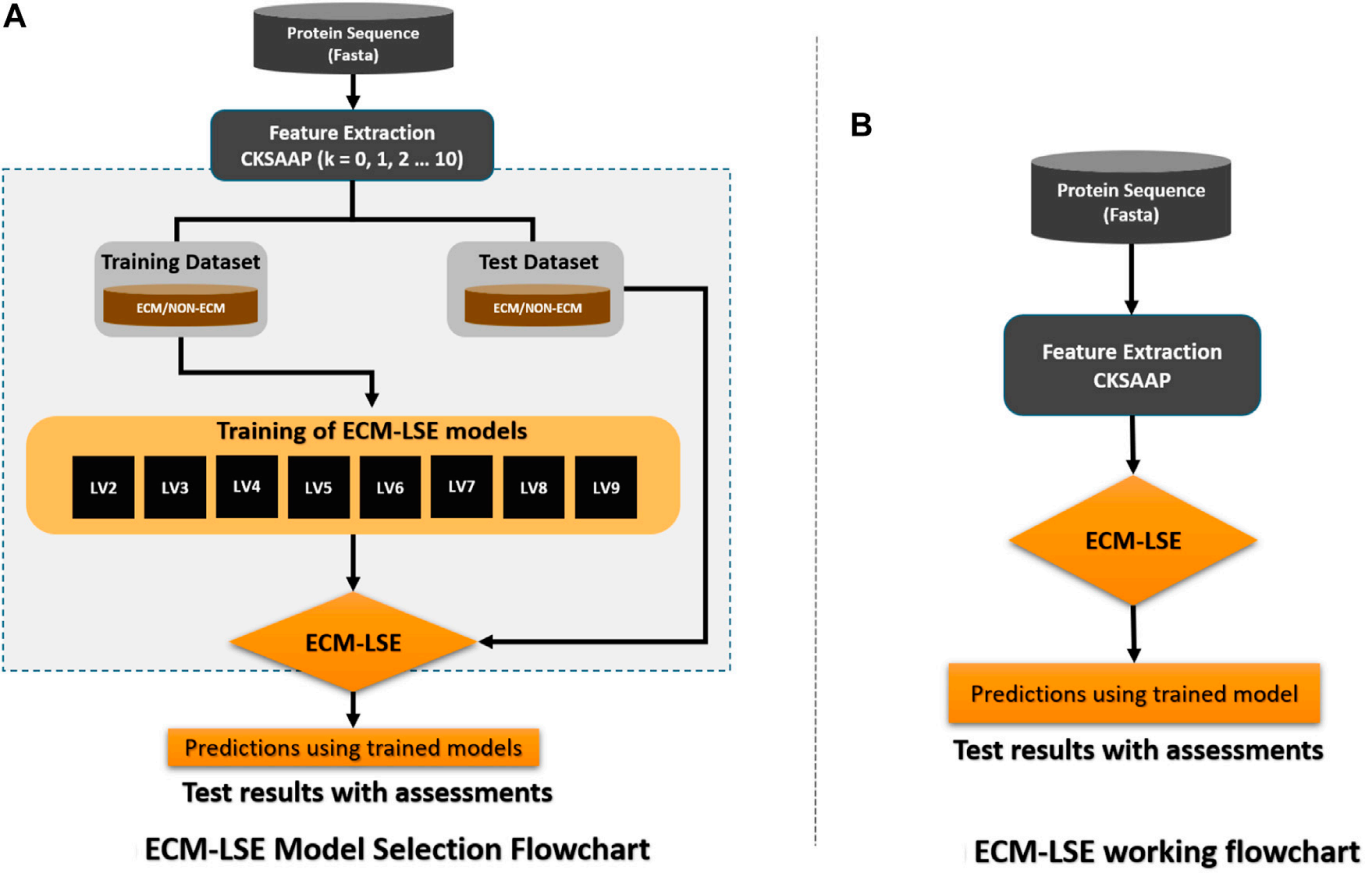
Workflow of the proposed ECM-LSE method.

**FIGURE 4:**
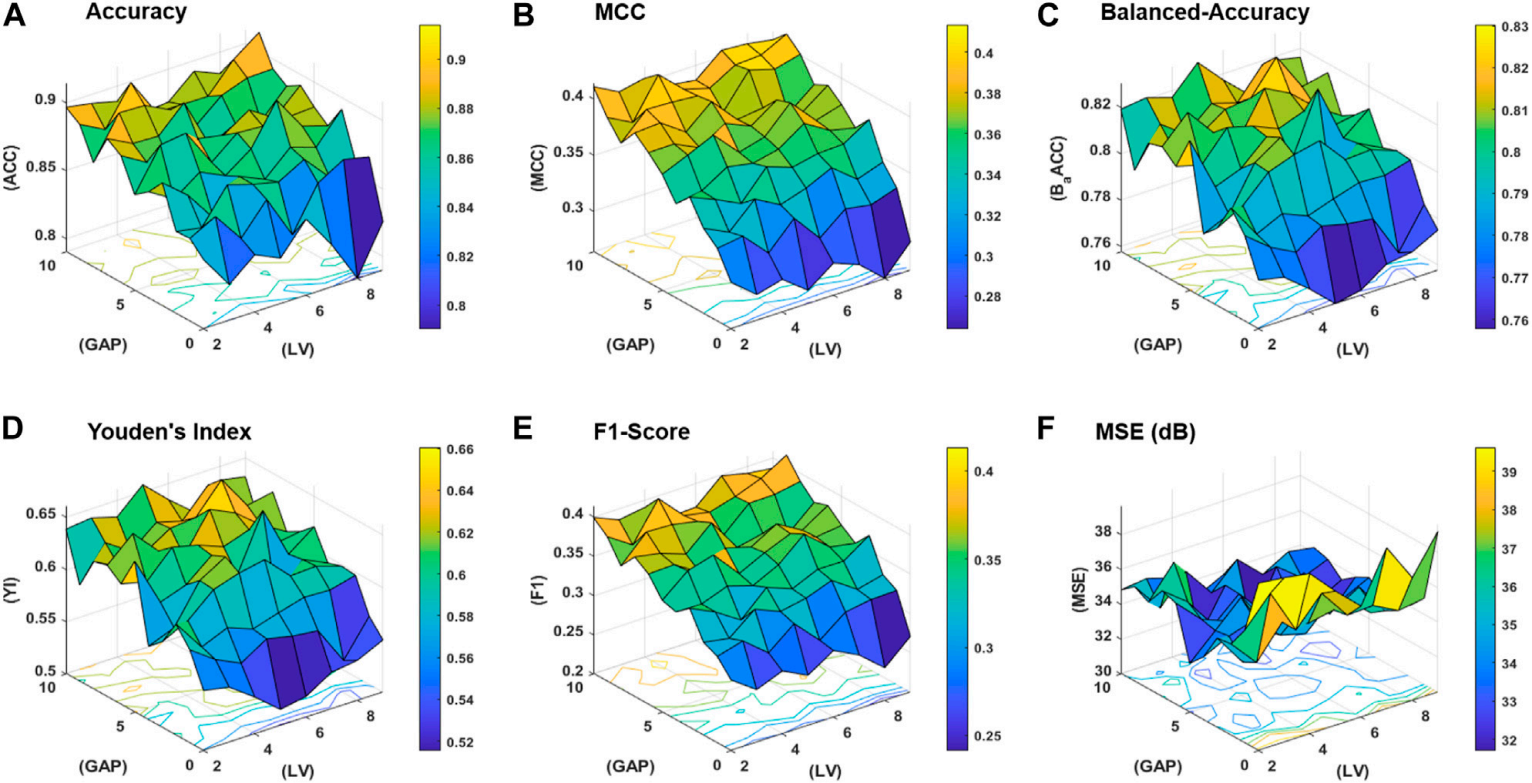
Performance statistics surfaces for: **(A)** accuracy, **(B)** MCC, **(C)** balanced-accuracy, **(D)** Youden’s Index, **(E)** F1-Score, and **(F)** mean squared error (MSE) in dB.

The workflow of the proposed study is aimed to obtain the best classification model based on two variables, that is, the gap between the two amino acid pairs and the number of units in the latent space *LVs*. An ablation study has been designed to acquire models with varying number of aforementioned variables and is depicted in Figure 3 (a). The samples are distributed into training, validation, and test datasets as discussed in the Subsection 2.2 and are encoded with incrementing values of *k* from 0 to 10. The resultant features are used to train the model with incrementing values of the latent space variables ranging from 2 to 9. As discussed earlier, for each configuration, 20 independent trials are performed and the mean results are computed. A consistent procedure is repeated for all 1,760 trials of the 88 unique model configurations. The model with the best average results is finally selected as the base model to perform prediction and is named as ECM-LSE. In Table. 1, the average results of the balanced accuracy have been reported. It can be observed that the model with values of gap *k* = 8 and latent variables *LV* = 7, accounts for the best. The results for the rest of the evaluation parameters are illustrated in the form of surface graphs in Figure 4.

**TABLE 1:** Balanced accuracy results of ablation study on Gap (*k*) and *LV* parameters.

Gap/*LV*	2	3	4	5	6	7	8	9
*k* = 0	0.779 ± 0.022	0.776 ± 0.027	0.768 ± 0.026	0.758 ± 0.034	0.760 ± 0.028	0.767 ± 0.015	0.769 ± 0.029	0.775 ± 0.027
*k* = 1	0.795 ± 0.025	0.786 ± 0.030	0.780 ± 0.027	0.788 ± 0.020	0.785 ± 0.030	0.784 ± 0.038	0.765 ± 0.034	0.783 ± 0.030
*k* = 2	0.803 ± 0.021	0.788 ± 0.036	0.793 ± 0.025	0.789 ± 0.029	0.793 ± 0.024	0.796 ± 0.030	0.795 ± 0.021	0.798 ± 0.027
*k* = 3	0.791 ± 0.031	0.797 ± 0.029	0.808 ± 0.015	0.812 ± 0.018	0.814 ± 0.028	0.803 ± 0.027	0.803 ± 0.030	0.799 ± 0.32
*k* = 4	0.785 ± 0.028	0.790 ± 0.047	0.809 ± 0.021	0.816 ± 0.026	0.797 ± 0.029	0.786 ± 0.026	0.803 ± 0.021	0.797 ± 0.037
*k* = 5	0.822 ± 0.018	0.799 ± 0.032	0.803 ± 0.035	0.813 ± 0.025	0.800 ± 0.031	0.826 ± 0.021	0.802 ± 0.023	0.811 ± 0.019
*k* = 6	0.808 ± 0.046	0.805 ± 0.023	0.817 ± 0.021	0.814 ± 0.026	0.810 ± 0.027	0.814 ± 0.022	0.803 ± 0.021	0.805 ± 0.031
*k* = 7	0.813 ± 0.032	0.824 ± 0.033	0.812 ± 0.029	0.806 ± 0.024	0.824 ± 0.027	0.818 ± 0.029	0.808 ± 0.041	0.801 ± 0.022
*k* = 8	0.811 ± 0.029	0.805 ± 0.039	0.807 ± 0.034	0.815 ± 0.021	0.816 ± 0.021	0.830 ± 0.021	0.814 ± 0.029	0.816 ± 0.026
*k* = 9	0.796 ± 0.034	0.813 ± 0.022	0.804 ± 0.029	0.814 ± 0.026	0.811 ± 0.034	0.824 ± 0.032	0.809 ± 0.025	0.798 ± 0.034
*k* = 10	0.819 ± 0.037	0.821 ± 0.021	0.817 ± 0.034	0.823 ± 0.021	0.817 ± 0.027	0.807 ± 0.025	0.819 ± 0.025	0.816 ± 0.031

### 3.2 Comparison With Contemporary Approaches

The performance of the proposed model is compared to the benchmark approaches and the findings are reported in Table 2. For a fair comparison, only the best reported results of the respective approaches are presented. The performance of the proposed ECM-LSE is compared with the contemporary methods including EcmPred (Kandaswamy et al., 2013), a sparse learning approach for the prediction of ECM (ECMSRC) (Naseem et al., 2017), and PECM (Zhang et al., 2014). In particular, the reported sensitivity, specificity, MCC, Youden’s index, and accuracy on the benchmark dataset of EcmPred (Kandaswamy et al., 2013) are compared.

**TABLE 2:** Comparison of the proposed ECM-LSE algorithm with the benchmark machine learning approaches on the test dataset.

Method	Sensitivity (%)	Specificity (%)	MCC	Youden’s index	Accuracy (%)	Balanced accuracy (%)
EcmPred (Kandaswamy et al., 2013)	65.00	77.00	0.1910	0.42	77.00	71.00
ECMSRC (Naseem et al., 2017)	74.48	81.31	0.2560	0.56	81.06	77.90
PECM (Zhang et al., 2014)	75.86	**86.88**	0.3143	0.63	**86.52**	81.37
**ECM-LSE**	**84.14**	86.45	**0.3906**	**0.71**	86.35	**85.30**

Bold-face represent best performance.

The results clearly show that the proposed method has better balanced accuracy as compared to the contemporary approaches. In particular, the proposed ECM-LSE method achieves the highest sensitivity of 84.14*%* outperforming the best competitor (PECM) by a margin of 10.91*%*. The specificity value achieved by the proposed ECM-LSE also compares favorably with other methods, which confirms the balanced unbiased learning effect. It is noteworthy to point out that the accuracy metric cannot provide true fitness of the models given the skewed distribution of test dataset toward the negative (non-ECM) class. Any model with all negative predictions can achieve $100\times \frac{2247}{145+2247}=93.94\%$ accuracy easily. As discussed in Subsection 2.1, the parameters of balanced accuracy, MCC, and Youden’s index are considered more reliable in the case of imbalanced dataset. Therefore, despite achieving 86.35*%* test accuracy, which is 0.17*%* lower than the PECM, better balanced accuracy and Youden’s index values, which is 3.93*%* and 0.08 units higher, respectively, demonstrate the superiority of the proposed method. Similarly, the MCC value achieved by ECM-LSE is 7.63*%* higher than the PECM method. MCC metric is preferred for accuracy and is considered as more reliable statistical parameter because it produces a higher value only if the classifier achieved good results in all four categories of the confusion matrix (Chicco and Jurman, 2020). In general, the proposed ECM-LSE approach has shown to comprehensively outperform the contemporary methods in all aspects of balanced and unbiased prediction performance.

Furthermore, unlike contemporary methods where handcrafted embedding schemes are utilized for separately developing the classifier and the feature extraction modules, the proposed ECM-LSE method learns directly from the original feature space. The LSE encoding effectively learns the distinguishing characteristics of classes in a lower dimensional feature space and allows the visualization of proteins sequences. This aspect of ECM-LSE is further explained in Section 3.4.

### 3.3 Verification on Experimentally Verified Human ECM Proteins

To verify the practical usefulness of our method, herein, we perform the validation of our method on experimentally verified ECM proteins. In particular, we collected 20 experimentally verified human ECM proteins from UniProt (Consortium, 2018). The collected sequences were not present in the positive or negative datasets of ECM-LSE. The criteria for the selection were based on the clear experimental evidence in the literature for the given sequence entry. We evaluated the EcmPred (Kandaswamy et al., 2013), ECMSRC (Naseem et al., 2017), PECM (Zhang et al., 2014), and ECM-LSE methods. As shown in Table 3, ECM-LSE (*k* = 8 and *LV* = 7) correctly identified 19 proteins as extracellular matrix proteins, whereas PECM, ECMSRC, and EcmPred identified 18, 16, and 15 proteins, respectively. It is noteworthy to point out that the models were trained on ECM proteins from metazoans; therefore, the superior performance of the proposed ECM-LSE on proteins from a completely different organism suggests that it can be effectively utilized for the annotation of unknown proteins.

**TABLE 3:** Prediction results for 20 experimentally verified ECM proteins. “***✔***” indicates correctly identification while “✗” represents an incorrect identification.

UniProtKB ACC	NCBI definition	EcmPred	ECMSRC	PECM	ECM-LSE
Q9BY76	Angiopoietin-related protein	***✔***	*✔*	*✔*	*✔*
P07355	Annexin A2	*✔*	*✔*	*✔*	*✔*
Q9BXN1	Asporin	*✔*	*✔*	*✔*	*✔*
P01137	Transforming growth factor beta-1	✗	✗	*✔*	*✔*
Q8N6G6	ADAMTS-like protein 1	*✔*	*✔*	*✔*	*✔*
P27797	Calreticulin	*✔*	*✔*	*✔*	*✔*
Q76M96	Coiled coil domain–containing protein	*✔*	*✔*	*✔*	*✔*
Q07654	Trefoil factor 3	✗	*✔*	✗	*✔*
O75339	Cartilage intermediate layer protein 1	*✔*	*✔*	*✔*	*✔*
Q15063	Periostin	✗	✗	*✔*	*✔*
O43405	Cochlin	*✔*	*✔*	*✔*	*✔*
Q96P44	Collagen alpha-1(XXI) chain	*✔*	*✔*	*✔*	*✔*
P01009	Alpha-1-antitrypsin	✗	*✔*	✗	✗
Q14118	Dystroglycan	*✔*	✗	*✔*	*✔*
Q12805	EGF-containing fibulin-like extracellular matrix protein 1	*✔*	*✔*	*✔*	*✔*
Q75N90	Fibrillin-3	*✔*	*✔*	*✔*	*✔*
P09382	Galectin-1	*✔*	*✔*	*✔*	*✔*
Q8N2S1	Latent-transforming growth factor beta–binding protein 4	*✔*	*✔*	*✔*	*✔*
P27487	Dipeptidyl peptidase 4	✗	✗	*✔*	*✔*
P08253	72 kDa type IV collagenase	*✔*	*✔*	*✔*	*✔*

### 3.4 Discussion

**FIGURE 5:**
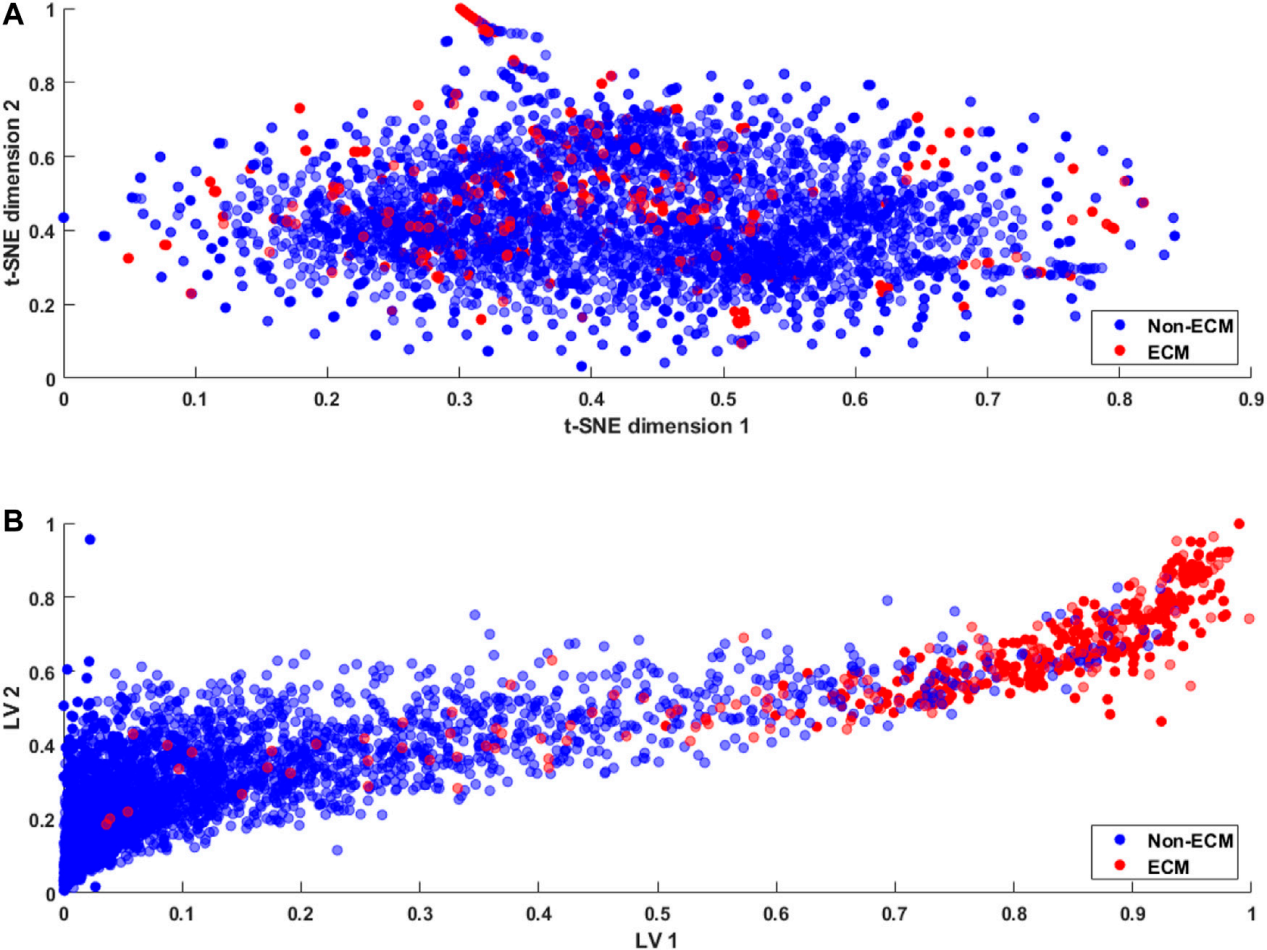
Feature embedding **(A)** using t-SNE (Van der Maaten and Hinton, 2008) projection of the original feature space and **(B)** using the proposed latent space encoding (ECM-LSE) method.

For typical classification problems such as lysine acetylation site prediction in proteins (Wu et al., 2019) or the identification of protein–protein binding sites (Fernandez-Recio et al., 2005), a large number of positive and negative samples are usually available in the datasets. Therefore, the problem of class imbalance or intra-class variation is not a major concern (Johnson and Khoshgoftaar, 2019). However, the limited availability of ECM samples results in an imbalanced dataset, resulting in an ill-posed problem. A number of approaches, including sample rescaling, have been proposed in the literature to tackle the imbalanced data problem (Xiao et al., 2016; Kabir et al., 2018). Classifiers based on these rescaling techniques tend to behave well; however, the generalization of the method is compromised. Furthermore, the comparison of methods using rescaled samples with the methods using a standard dataset is not reasonable. In the proposed study, we utilize a standard dataset and develop a method that effectively discriminates the ECM proteins from non-ECM. This is achieved through the latent space learning of the CKSAAP features. For better understanding, we compare the t-SNE projection of the CKSAAP features with the proposed latent space in Figure 5.

For visualization purposes, the data were projected on two dimensions using t-SNE (Van der Maaten and Hinton, 2008) projection of the original feature space and two variable latent spaces in the case of ECM-LSE. In the t-SNE projection shown in Figure 5A, it can be observed that both ECMs and non-ECMs appear in an overlapping fashion, suggesting that the development of the ECM classifier using original feature space is an arduous task. As shown in Figure 5B, the proposed latent space encoding (ECM-LSE) presents superior learning capabilities and maps the ECMs and non-ECMs in separate regions in contrast to the unsupervised subspace learning method of t-SNE (Van der Maaten and Hinton, 2008).

The proposed method, as shown in Figure 5B, tends to form distinguishable clusters of ECM and non-ECM proteins. Although some overlap can be observed in the projection of the proposed method, it is still remarkably better than that of the t-SNE, and since the projection is shown for two latent variables only, the actual model with seven latent variables is expected to mitigate the overlap to a greater extent. These projections are also helpful in understanding the working principle of the proposed method and the motivation for the development of nonlinear auto-encoded learning of latent space.

The proposed hybrid approach presents a hybrid design with capabilities of efficient feature selection and classification of ECM proteins. The latent space dynamically reduces the dimension of the feature space and retains only the relevant information sufficient to efficiently distinguish ECM from non-ECM samples. Although, the proposed method can predict ECM from different organisms, it is not a replacement for gold standard wet lab–based testing. Furthermore, due to the scarcity of available ECM proteins the model may show biased performance in favor of already explored ECM and finding novel proteins may require the fusion of additional information. However, efforts have been made to avoid overfitting in order to seek the generalization property of the model by deploying dropout and batch normalization techniques. Further enhancements to the ECM prediction task where scarcity of the positive samples persists can be made by applying a transfer learning approach, where a large scale model is trained on a closely related dataset and is further fine-tuned for ECM samples. The Python implementation of the proposed algorithm has been made public, and interested users can utilize the algorithm for their problem of interest. The algorithm is available at (https://github.com/Shujaat123/ECM-LSE/blob/master/ECM_LSE_Online.ipynb). In the future, we aim to explore the efficacy of the auto-encoder–based classifiers on other bioinformatics problems.

## 4 Conclusion

ECM is a complex meshwork of cross-linked proteins responsible for the architectural support of cells and contributes to the functionality of the living tissue. They also contribute toward the formation of the cancer stem cells; therefore, their study and classification from non-ECMs proteins is of prime importance. A reliable prediction method can not only help understand various abnormalities associated with several cancer types but will also assist in diagnostic research. Conventional experimental-based methods are considered gold standards for this task; however, they are extremely time consuming and scanning a large number of proteins is practically infeasible. In this research, we designed a latent space learning method for the classification of ECM proteins. The proposed method can be used as a reliable prediction model. An important feature of the proposed method is its latent space-based projections through which protein sequences can be visualized in filtered and reduced dimensions, which is extremely helpful in finding useful clusters. The proposed method has been tested on a benchmark dataset and results of widely used performance metrics are reported. In particular, we report a balanced test accuracy of 86.45*%* with 0.71 Youden’s index and 0.39 MCC (with *k* = 8 and *LV* = 7). Additionally, the model performance is verified on completely unseen experimentally verified ECM proteins and shown to achieve highest prediction score.

## Data Availability

Publicly available datasets were analyzed in this study. This data can be found here: https://github.com/Shujaat123/ECM-LSE.
